# Discovery of novel papillomaviruses in the critically endangered Malayan and Chinese pangolins

**DOI:** 10.1098/rsbl.2022.0464

**Published:** 2023-01-04

**Authors:** Jose Gabriel Nino Barreat, Anselmo Jiro Kamada, Charles Reuben de Souza, Aris Katzourakis

**Affiliations:** Department of Biology, University of Oxford, Oxford OX1 2JD, UK

**Keywords:** *Papillomaviridae*, *Manis javanica*, *Manis pentadactyla*

## Abstract

Pangolins are scaly and toothless mammals which are distributed across Africa and Asia. Currently, the Malayan, Chinese and Philippine pangolins are designated as critically endangered species. Although few pangolin viruses have been described, their viromes have received more attention following the discovery that they harbour sarbecoviruses related to SARS-CoV-2. Using large-scale genome mining, we discovered novel lineages of papillomaviruses infecting the Malayan and Chinese pangolins. We were able to assemble three complete circular papillomavirus genomes with an intact coding capacity and five additional L1 genes encoding the major capsid protein. Phylogenetic analysis revealed that seven out of eight L1 sequences formed a monophyletic group which is the sister lineage to the Tupaia belangeri papillomavirus 1, isolated from Yunnan province in China. Additionally, a single L1 sequence assembled from a Chinese pangolin was placed in a clade closer to *Alphapapillomavirus* and *Omegapapillomavirus*. Examination of the SRA data from 95 re-sequenced genomes revealed that 49.3% of Malayan pangolins and 50% of Chinese pangolins were positive for papillomavirus reads. Our results indicate that pangolins in South-East Asia are the hosts of diverse and highly prevalent papillomaviruses, and highlight the value of *in silico* mining of host sequencing data for the discovery of novel viruses.

## 1. Introduction

Pangolins are scaly, primarily nocturnal and insectivorous mammals which belong to the order Pholidota. They are classified into three genera: two are found in Africa (*Smutsia*, *Phataginus*), and one in Asia (*Manis*) [1]. There are four different species of pangolins in Asia: the Chinese pangolin (*Manis pentadactyla*), the Malayan pangolin (*Manis javanica*), the Indian pangolin (*Manis crassicaudata*) and the Philippine pangolin (*Manis culionensis*) [1]. The Chinese, Malayan and Philippine pangolins are currently designated as critically endangered species by the International Union for the Conservation of Nature (IUCN), due to their population decline as a result of overexploitation and trafficking for their scales [2–4].

Pangolins have recently gained attention as a potential host of emerging viral diseases after coronaviruses related to SARS-CoV-2 were reported in the Malayan pangolin [5–10]. Pangolins have also been recognized as hosts for other RNA viruses such as canine distemper virus (*Paramyxoviridae*), which is associated with respiratory, digestive and neurological illness in pangolins [11], flaviviruses, reoviruses, pneumoviruses and picornaviruses [12–15]. Overall, the diversity of DNA viruses and their disease association in pangolins is less well known; so far anellovirus, parvovirus, circovirus and genomovirus genomes have been described [15,16].

Papillomaviruses are non-enveloped, dsDNA viruses with a circular genome, which can cause a diverse array of clinical manifestations in their vertebrate hosts, ranging from subclinical, to cutaneous and mucosal warts, and cancerous lesions [17]. We describe the discovery of two novel lineages of papillomaviruses found by mining the genome data of the Malayan and Chinese pangolins. These findings constitute the first detailed record of papillomavirus infection in pangolins and highlight the need for a systematic assessment of the diversity and biology of DNA viruses hosted by these animals.

## 2. Methods

We discovered an unidentified papillomavirus contig (NW_023450026.1) in pangolins by querying the RefSeq eukaryotic genomes database (ref_euk_rep_genomes) with 1413 papillomavirus reference proteins obtained from the NCBI Virus Resource (June/2022) [18,19]. Screening was performed using the tblastn algorithm (-task tblastn-fast) implemented by the ElasticBLAST (v0.2.6) method on the Google Cloud Platform [20,21]. The search returned 1017 hits with *e*-values less than 1×10^−5^ to an unplaced genomic scaffold (YNU_ManJav_2.0 scaffold_14136) from the RefSeq genome assembly of the Malayan pangolin.

The 7307-bp contig was annotated using the PuMA pipeline [22]. This sequence corresponded to a full papillomavirus genome encoding L1, L2, E1, E2, E6 and E7, in addition to two spliced products (E1^E4 and E8^E2). Given that the papillomavirus genome was intact, we screened the short-read data of the re-sequenced genomes of 72 Malayan pangolin and 22 Chinese pangolin individuals, which were deep-sequenced from samples of pangolin muscle by Hu *et al*. [23]. We obtained the SRA experiment accession numbers from this study (BioProject IDs: PRJNA529540 and PRJNA529512) and used a combination of blastn and tblastn on the NCBI [24] to find reads with significant similarity to papillomaviruses.

We downloaded the short-read sequences of the SRA experiments with more than 100 significant matches (*e*-value < 0.01) and tried to *de novo* assemble complete viral genomes or the L1 gene. Fasta files were concatenated, and the duplicate sequences were removed with SeqKit [25]. We used a custom Python 3 script to sort the sequences into forward, reverse and orphan reads (script available in the electronic supplementary material). The reads in these files were assembled in SPAdes v3.15.4 [26], using the ‘–metaviral’ and ‘–assembler-only’ flags. Contigs of complete viral genomes were annotated using PuMA. Contigs of L1 genes were examined in ORFfinder to check for the presence of an intact L1 open reading frame [27]. The molecular weight and isoelectric point of predicted protein products were estimated in ExPASy [28]. We calculated the coverage (depth) of our assembled sequences using Magic-BLAST [29], mapped reads were sorted and the coverage calculated using samtools [30].

To study the systematics of these viruses, we inferred a Bayesian phylogeny of the L1 and E1 proteins. We first selected a set of viruses using the pangolin papillomavirus L1 sequences in searches of the PaVE papillomavirus taxonomy tool [31]. We chose papillomaviruses recognized by the ICTV in addition to the Tupaia belangeri papillomavirus 1 (TbelPV1) and Tupaia belangeri papillomavirus 2 (TbelPV2) described in the study of Liu *et al*. [32]. Sequences were aligned in MAFFT v7.490 using the accurate option (MAFFT L-INS-i) [33]. Alignments were trimmed and the best substitution models for the alignments (both LG + I + G4 + F) were found in ModelTest-NG [34]. We then inferred a Bayesian phylogeny in MrBayes version 3.2.7a [35] with an MCMC chain length of 1 000 000 or 4 000 000 generations, respectively (burn-in = 25%). In both cases, convergence was assessed by ensuring that the average s.d. of split frequencies was less than 0.01, and the potential scale reduction factor for all parameters was approximately 1.

## 3. Results

We found significant hits to papillomaviruses in 36 out of 73 (49.3%) samples of the Malayan pangolin, and 11 out of 22 (50%) samples from the Chinese pangolin (table 1; electronic supplementary material, table S1). All the samples with known geographical origin came from the Yunnan province of China (sampled between the years 2000 and 2005). The remaining 42 individuals were seized in Yunnan (years 2016–2017) or at the border between Yunnan and Myanmar (year 2014). From the positive individuals, 10 out of 73 (13.7%) Malayan pangolins, and one out of 22 (4.6%) Chinese pangolins had more than 100 significant hits. We selected these for assembly of the complete genome/L1 gene. Three complete genomes were assembled *de novo*: two new papillomavirus genome assemblies (MJ55: depth = 36.71*x*, MJ23: depth = 10.22*x*) and the reassembled genome for the reference contig (MJ74: depth = 13.06*x*). We could also assemble the complete L1 gene for three other individuals (MJ18, MJ33 and MP15). Interestingly, three different L1 gene contigs were assembled from a single Chinese pangolin individual, designated as MP15A, MP15B and MP15C.

**Table 1.** Summary of the SRA experiments from pangolins with greater than 70 significant hits (blastn, *e*-value < 0.01) to the linearized papillomavirus genome of the reference individual MJ74. Sets of reads which could be assembled into a complete genome or complete L1 gene are indicated, with a reference to the mean depth (*x*) of the assembly. Samples with less than 70 positive reads are shown in the electronic supplementary material, table S1.

sample ID	seizure location	year	SRA accession	no. reads	% identity^a^	assembled sequence	depth (*x*)
MJ55	Yunnan, China	2016	SRR9018627	1239	77.47 [68.67–92.68]	complete genome	36.71
MP15	Yunnan, China	2016	SRR9018603	851	78.08 [68.06–100]	L1 (MP15A)	31.61
						L1 (MP15B)	7.50
						L1 (MP15C)	30.97
MJ74^b^	Yunnan, China	2014	SRR9005053	810	99.32 [71.96–100]	complete genome	13.06
			SRR9005054				
			SRR9005055				
			SRR9005056				
MJ23	Sino-Burmese border	2014	SRR9018636	342	79.03 [70.87–100]	complete genome	10.22
MJ33	Sino-Burmese border	2014	SRR9018645	311	79.17 [70.59–100]	L1	7.02
MJ44	Yunnan, China	2017	SRR9018615	208	77.11 [68.97–100]	—	—
MJ54	Yunnan, China	2017	SRR9018626	159	78.36 [68.57–100]	—	—
MJ12	Sino-Burmese border	2014	SRR9018668	135	79.65 [70.63–100]	—	—
MJ13	Yunnan, China	2017	SRR9018669	123	76.82 [69.06–88.46]	—	—
MJ59	Yunnan, China	2016	SRR9018621	117	77.5 [68.53–100]	—	—
MJ18	Sino-Burmese border	2014	SRR9018660	111	77.3 [69.92–87.90]	L1	6.18
MJ58	Yunnan, China	2016	SRR9018620	86	77.28 [70.25–87.50]	—	—
MP22	Yunnan, China	2016	SRR9018601	76	79.36 [70.20–88.46]	—	—
MJ30	Sino-Burmese border	2014	SRR9018646	73	77.47 [71.07–100]	—	—

^a^Pairwise identity of the reads to the query sequence expressed as mean [min.–max.].

^b^Genome reassembled from the SRA data (PRJNA529512) for the RefSeq reference sequence (NW_023450026.1).

**Figure 1.**
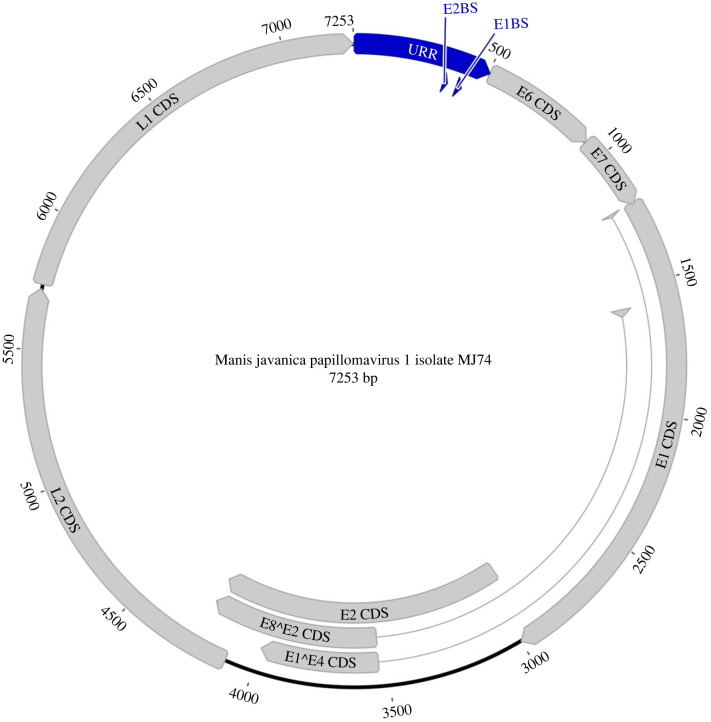
Complete papillomavirus genome assembled from the Malayan pangolin individual MJ74 and annotated with the PuMA pipeline. The 7253-bp circular genome encodes the four core papillomavirus proteins (L1, L2, E1 and E2) in addition to the E6 and E7 accessory proteins. Two spliced products were also identified: E1^E4 and E8^E2. The URR has also been annotated and includes the E1- and E2-protein binding sites (E1BS and E2BS). Image created in Geneious Prime 2022.2.1 [36].

The assembled genomes ranged in size from 7253 bp to 7437 bp with a GC content between 39.84 and 40.09%. All three genomes encode the four core papillomavirus proteins: E1, E2, L1 and L2, and two accessory proteins E6 and E7 (figure 1; electronic supplementary material, figures S1 and S2). We also identified two spliced products, E8^E2 and E1^E4 in two of the genomes. The splice donor and acceptor sequences were identified by homology in the PuMA tool [22]. The upstream regulatory regions (URRs) range in size from 504 to 613 bp. A table with the gene coordinates and features of the encoded proteins is provided in the electronic supplementary material, table S2.

**Figure 2.**
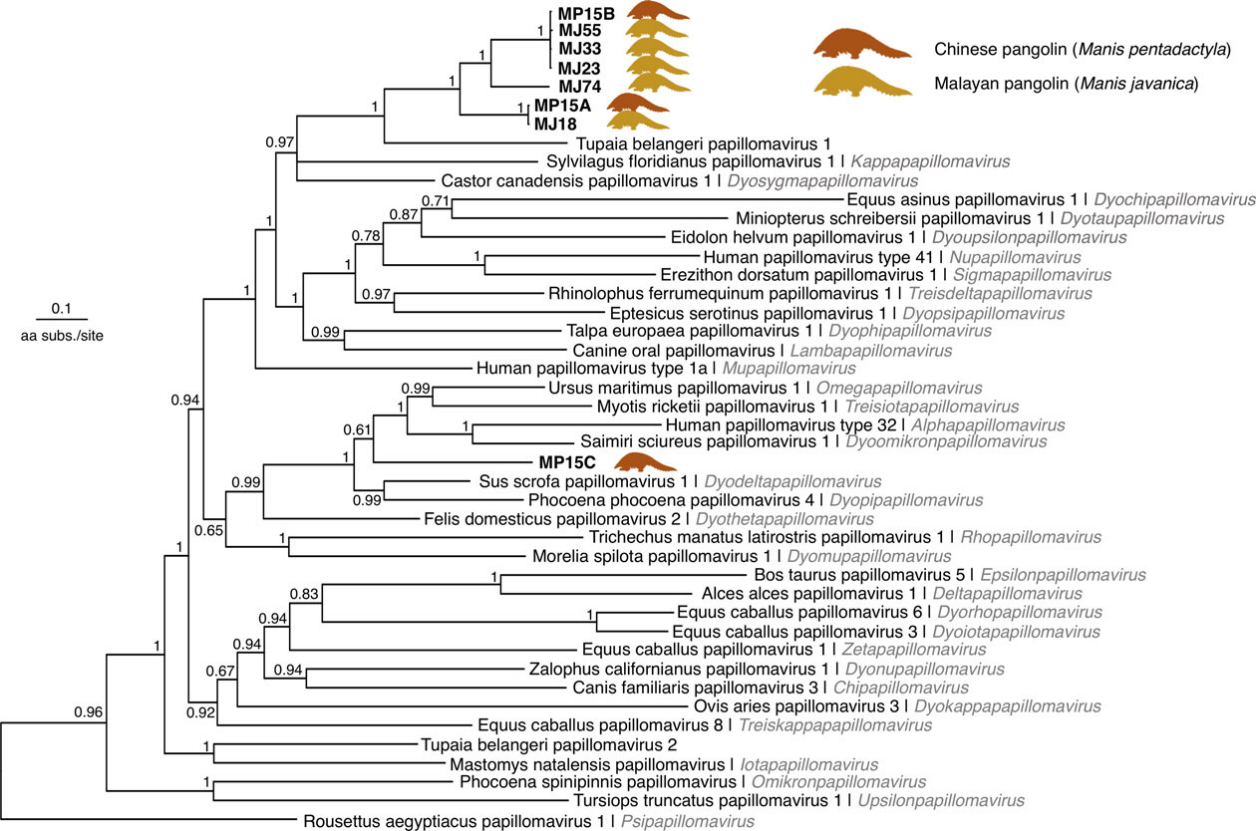
Bayesian phylogenetic tree of the L1 proteins of papillomaviruses. Most pangolin papillomavirus L1 sequences form a highly supported monophyletic group which is the sister to TbelPV1 (PP = 1). This clade includes sequences assembled from five Malayan pangolin individuals (MJ18, MJ23, MJ33, MJ55 and MJ74), and two sequences from a Chinese pangolin individual (MP15A and MP15B). A single sequence from the same Chinese pangolin individual (MP15C) was placed with high confidence (PP = 1) on a different clade with *Alphapapillomavirus*, *Omegapapillomavirus* and *Dyodeltapapillomavirus*. The tree was outgroup rooted with the L1 sequence from Sparus aurata papillomavirus 1 (*Secondpapillomavirinae*), which is not shown for clarity.

Phylogenetic analysis of the L1 proteins placed seven out of eight of the L1 sequences into a monophyletic group with high support (posterior probability, PP = 1) (figure 2). These sequences were grouped as the sister lineage to TbelPV1, also with high support (PP = 1). In the E1 phylogeny, this clade was also placed as the sister of TbelPV1 with high confidence (PP = 1) (electronic supplementary material, figure S3). A single L1 protein sequence from a Chinese pangolin (MP15C) was nested in a different clade that includes the genera *Omegapapillomavirus* isolated from polar bears, *Alphapapillomavirus* which infects primates and *Dyodeltapapillomavirus* which infects domestic pigs (PP = 1). However, the position of the sequence within the clade had a low support (PP = 0.61), so its specific placement within this group is uncertain.

## 4. Discussion

We describe the complete genome for a novel papillomavirus, which constitutes the first detailed record of the family *Papillomaviridae* in pangolins. The assembly of complete circular genomes with an intact coding capacity, regulatory elements, and no host flanking sequences, shows that these are exogenous viruses highly likely to be infecting pangolins. In addition, finding these viruses across multiple individuals, sampled from different time-points and in two different host species, suggests the viruses naturally circulate in pangolin populations.

The demarcation criteria for papillomavirus species and genera are based on nucleotide identity thresholds across the L1 genes: viruses with greater than 70% identities are placed in the same species, and those with greater than 60% identities are placed in the same genus [17]. We found that seven out of eight of the predicted L1 protein sequences formed a highly supported clade, suggesting that this group belongs to a single species of papillomavirus present in the Malayan and Chinese pangolins (L1 nucleotide per cent identities: 71.87–99.93%). This clade, which we call Manis javanica papillomavirus 1 (MjavPV1), was placed as the sister group to TbelPV1, isolated from wild Northern tree shrews (*Tupaia belangeri*) in the Jianchuan and Lufeng localities of Yunnan province (years 2016–2017) [32]. Our results indicate that MjavPV1 belongs to the same (but still unnamed) genus as TbelPV1, given that the nucleotide per cent identities in the L1 gene are greater than 60% (63.22%–65.81%). The overlap in the ranges of the hosts in South-East Asia suggests a potential cross-species transmission may have occurred (electronic supplementary material, figure S4). This agrees with the L1 phylogeny where the papillomaviruses from more closely related hosts (e.g. tree shrews/primates and pangolins/carnivores) were placed in different clades (figure 2). In addition, the evolutionary pattern within pangolins suggests that MjavPV1 viruses have been exchanged between the two host species (figure 2). However, without further evidence, we cannot rule out the possibility of a deep co-divergence between the tree shrew and pangolin papillomaviruses.

Noticeably, we assembled two different L1 sequences (MP15A and MP15B), belonging to the MjavPV1 lineage from a single Chinese pangolin individual (MP15), along with another papillomavirus L1 sequence that clustered with *Omegapapillomavirus* and *Alphapapillomavirus* clade instead (figure 2, we refer to this virus provisionally as Manis pentadactyla papillomavirus 1, MpenPV1). However, taxonomic placement based on the identity of the L1 gene assigned MpenPV1 to the genus *Dyodeltapapillomavirus* (closest to Sus scrofa papillomavirus 1, identity = 66.85%) [31]. Therefore, the individual was likely co-infected by two distinct strains of MjavPV1, in addition to MpenPV1. These results suggest that pangolins in South-East Asia may potentially be the hosts of papillomaviruses from diverse lineages, which will require further study and characterization.

After examining the SRA samples for the re-sequenced genomes of 73 Malayan and 22 Chinese pangolins, we found positive reads for MjavPV1 in about 50% of individuals in both species. The sequences were confirmed to be of pangolin origin since they were sampled from muscle tissue of pangolins [23] (Y Li 2022, personal communication), they were assembled to a good coverage, show sequence variation, and have a unique phylogenetic placement in the papillomavirus phylogeny. However, as the samples were not collected specifically for microbiological examinations, we cannot rule out the presence of other tissues (e.g. epithelia), and so it remains unclear whether the viral tropism is specific towards muscle. Indeed, TbelPV1, which belongs to the same genus as MjavPV1, seems to have tropism for the oral mucosa [32]. In addition, finding papillomavirus (SRA) positive pangolin individuals distributed across a timespan of 17 years indicates that papillomavirus infections are prevalent among these pangolin species.

We also found reads with significant matches (*e*-values: 9⋅10^–29^–2⋅10^–3^) to the MjavPV1 genome in the samples from five individuals described in the meta-transcriptomic study of the Malayan pangolin by Shi *et al*. [15] (electronic supplementary material, table S3). The difference in the number of matching reads we obtained from the genomic and transcriptomic SRA samples may be attributed to latent papillomavirus infections and differences in sampling (different tissues were pooled together for the meta-transcriptomic work). We also examined an additional report of papillomavirus reads in the Malayan pangolin by Liu *et al*. [5]. However, we did not obtain any matches to our query sequences and found that the best-hits to their custom database were all human-HPV16 junctions (electronic supplementary material, table S4). No hits could be obtained using the reference HPV16 virus proteins in a tblastn, suggesting that the reads came from human DNA contamination.

We demonstrate the application of cloud-computing for the efficient mining of large-scale genomic datasets and the discovery of novel viral lineages. This data-driven virus discovery is expected to increase our knowledge of the virosphere and offer a springboard for the experimental characterization of viruses that have not been isolated or which silently infect their hosts [37]. Using these *in silico* screening methods, a new lineage of fish alloherpesvirus-like endogenous viruses has been discovered [38], in addition to millions of novel RNA viruses on a global scale [39–42]. Given the critically endangered conservation status of the Malayan and Chinese pangolins, it will be important to actively assess whether these viruses cause any disease or decrease the fitness of their hosts. It is also still unclear whether the Malayan pangolin populations in the islands of Borneo, Java and Sumatra, and the Philippine pangolin, also host similar papillomaviruses. Further studies into these questions may shed light on the impact papillomaviruses have on pangolins and inform potential strategies for conservation.

## Data Availability

The raw sequence data analysed in this work are publicly available under the BioProject identifiers: PRJNA529540, PRJNA529512. The assembled sequences are available in the Third Party Annotation section of the DDBJ/ENA/GenBank databases under the accession numbers TPA: BK062771-BK062778. All other data are available in the electronic supplementary material [43].
